# Structural and Dynamics Perspectives on the Binding of Substrate and Inhibitors in *Mycobacterium tuberculosis* DHFR

**DOI:** 10.3390/scipharm85030031

**Published:** 2017-09-15

**Authors:** Pimonluck Sittikornpaiboon, Pisanu Toochinda, Luckhana Lawtrakul

**Affiliations:** School of Bio-Chemical Engineering and Technology, Sirindhorn International Institute of Technology, Thammasat University, Pathum Thani 12121, Thailand; pimonluck@hotmail.com (P.S.); pisanu@siit.tu.ac.th (P.T.)

**Keywords:** molecular dynamics simulations, antifolates, protein-ligand interactions

## Abstract

Dihydrofolate reductase (DHFR), an essential enzyme in the folate pathway, is a potential target for new anti-tuberculosis drugs. Fifteen crystal structures of *Mycobacterium tuberculosis* DHFR complexed with NADPH and various inhibitors are available in the RCSB Protein Data Bank, but none of them is a substrate binding structure. Therefore, we performed molecular dynamics simulations on ternary complexes of *M. tuberculosis* DHFR:NADPH with a substrate (dihydrofolate) and each of three competitive inhibitors in 2,4-diaminopyrimidine series (P1, P157, and P169), in order to gain insight into the inhibition-mechanism of DHFR in the folate pathway. The binding energy and thermodynamics values of each system were calculated by the Molecular Mechanics/Generalized Born Surface Area (MM/GBSA) method. The dynamics of the enzyme and the motion of each amino acid residue at the active site were examined. The key factors that promote the binding of P157 and P169 on *M. tuberculosis* DHFR (mtbDHFR) reveal opportunities for using these compounds as novel anti-tuberculosis drugs.

## 1. Introduction

Tuberculosis (TB) is an infectious disease caused by *Mycobacterium tuberculosis*. Treatment of TB requires taking a combination of drugs for at least six to nine months. Due to the long treatment time, improper use of these drugs can promote the development of drug-resistant *M. tuberculosis* strains. In 2015, the World Health Organization reported that 1.4 million people died from multidrug-resistant TB (MDR-TB) with up to 480,000 new cases of MDR-TB globally [1]. According to the rapid growth of MDR-TB and the fatality of the disease, the development of high efficacy drugs for TB is urgently needed.

Dihydrofolate reductase (DHFR) is a notable drug target for the design of anti-malarial [2], anti-bacterial [3], and anti-cancer [4] drugs. DHFR is an enzyme necessary for the folate biosynthesis pathway in eukaryotic and prokaryotic cells [5,6,7,8]. The enzyme DHFR catalyzes the reduction of 7,8-dihydrofolate (DHF) to the product 5,6,7,8-tetrahydrofolate (THF) by hydride transfer from the NADPH cofactor. The product THF is a precursor for the synthesis of RNA, DNA, and protein to promote the cell growth and proliferation of living organisms [9]. Mostly, inhibitors of DHFR inhibit the enzyme by competitive inhibition, which involves binding to the active site of a substrate, specifically, a non-allosteric site. However, few cases of inhibitor are bind to allosteric site on the enzyme surface [10]. A pocket-based virtual ligand screening (VLS) tool, PoLi, revealed that a unique scaffold of ononetin occupies the unique site on *Escherichia coli* DHFR [11]. Dias’s group reported the binding affinity of three antifolate drugs (pyrimethamine, cycloguanil, and trimethoprim) for *M. tuberculosis* DHFR (mtbDHFR). These three drugs bind to the active site of the substrate, and pyrimethamine (P1), which is a 2,4-diaminopyrimidine compound, exhibits the highest binding affinity for mtbDHFR [12].

**Figure 1:**
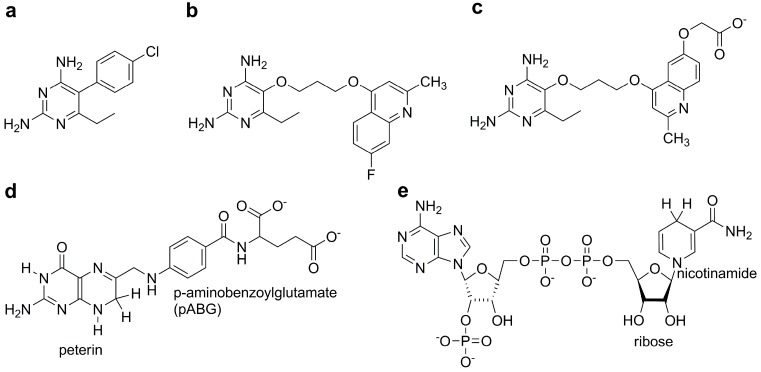
Chemical structures of three compounds; (**a**) P1; (**b**) P157; and (**c**) P169; substrate; (**d**) Dihydrofolate (DHF), and cofactor; (**e**) (NADPH).

In our previous work, molecular docking calculations of 50 2,4-diaminopyrimidine derivatives classified compounds into low affinity and high affinity groups against mtbDHFR [13]. However, their inhibition-mechanism with DHFR in the folate pathway and the dynamic motions of enzymes upon the binding of different ligands in an aqueous environment are not fully understood. Dynamics of the enzyme, especially the fluctuation of Met20 loop, play an important role in enzyme catalysis pathway of *E. coli* DHFR [14]. Sawaya and Kraut (1997) reported a structure of *E. coli* DHFR during the catalytic pathway and explained the motion of the loop and subdomain movement. The loop Met20 is closed in the holoenzyme (DHFR:NADPH), and the Michaelis complex (DHFR:NADPH:DHF) while the other complexes show an occluded conformation [15]. In closed conformation, the Met20 loop is moved closer to the active site and forces the nicotinamide and pterin rings to stay close together in the active site of *E. coli* DHFR [16]. For mtbDHFR, the Met20 loop is equivalent to L1 [12]. The motions of mtbDHFR are needed in order to understand the different binding affinities of inhibitor and substrate to the enzyme. There are 15 crystal structures of *M. tuberculosis* DHFR complexed with NADPH and various inhibitors in the RCSB Protein Data Bank (PDB; http://www.rcsb.org) [17], but none of them is a substrate binding structure. Therefore, we performed molecular dynamics (MD) simulations on ternary complexes of mtbDHFR:NADPH, with substrate (DHF) and each of the three 2,4-diaminopyrimidine compounds (P1, P157, and P169) as inhibitors, in order to gain insight into the catalytic-mechanism and inhibition-mechanism of DHFR in the folate pathway with implicit water. Chemical structures of ligands, bound to mtbDHFR in this study, are shown in Figure 1.

The binding energy (ΔG) and thermodynamics values of each system were calculated by the Molecular Mechanics/Generalized Born Surface Area (MM/GBSA) method [18]. The dynamics of the enzyme and the motion of each amino acid residue at the active site, which may play a role in ligand binding, were examined.

## 2. Method of Calculations

### 2.1. Molecular Structure Preparations

The crystal structure of the ternary complex of mtbDHFR:NADPH with P1 (PDB ID: 4KM0) was downloaded from the RSCB Protein Data Bank [17]. All missing hydrogen atoms were added by the Discovery Studio Visualizer 4.0 program [19]. The initial conformations of three compounds (P1, P157, and P169) and the substrate (DHF) were built by using GaussView 05 [20]. Further geometry optimizations were conducted by the density functional theory at the B3LYP/6-31G(d,p) level, as included in the Gaussian 09 program package [20].

### 2.2. Molecular Modeling Studies

Starting coordinates of the ternary complex of mtbDHFR:NADPH with each of P1, P157, P169, and DHF were modeled by molecular docking calculations using the AutoDock 4.2 software package [21]. The most stable conformation with the lowest binding energy and the highest percent frequency of each protein-ligand complex was selected to perform MD simulations in aqueous solution by using the AMBER12 software package [22]. The protein and ligands were parameterized according to the ff12SB force field [23] and general AMBER force field [24], respectively. Systems were solvated by the TIP3P water model [25] in a periodic truncated octahedral box with a density of around 1 g/cm^3^. Na^+^ counter ions were added to neutralize the system. After system energy minimization, the systems were heated from 0 K to 300 K over 20 ps. MD simulations were run for 20 ns at 300 K and 1 atm. The SHAKE algorithm [26] for bond constraints and the Langevin thermostat for temperature control were also applied.

### 2.3. Thermodynamics Quantities Calculations

The binding energy (ΔG_bind_) of complexation was calculated using Equation (1).

ΔG_bind_ = ΔH − TΔS = ΔE_ele_ + ΔE_vdw_ + ΔG_GB_ + ΔG_SA_ − TΔS (1)

where ΔH and entropy term (TΔS) are the enthalpy change, and the temperature with the conformational entropy change upon protein and ligand binding, respectively. The enthalpy change is calculated from the summation of electrostatic energy (ΔE_ele_), van der Waals energy (ΔE_vdw_), electrostatic solvation energy or polar contribution (ΔG_GB_), and non-electrostatic solvation component or non-polar contribution (ΔG_SA_).

Five thousand conformations were collected from the last 8 ns of the MD simulation of each system to calculate the ΔG_bind_. Moreover, a pairwise energy decomposition was also achieved to monitor the important residues involved in the interactions between the enzyme and ligand.

## 3. Results and Discussion

### 3.1. Equilibration of MD Simulations

**Figure 2:**
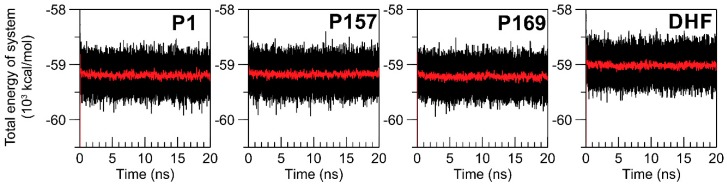
Total energy (E_total_) of ternary *M. tuberculosis* DHFR (mtbDHFR)-NADPH complexed with P1, P157, P169, and DHF during 20 ns of molecular dynamics (MD) simulations (black lines) and the average values of E_total_ in each 20-ps time interval during the 20-ns MD simulations (red lines).

To monitor the stability of the systems, the total energy (E_total_) during 20 ns of MD simulations was investigated (Figure 2). The average values of E_total_ in each period of 20 ps are also presented (Figure 2, red lines), which were practically stable throughout the simulations, around −59 × 10^3^ kcal/mol, indicating the equilibrium of the systems.

### 3.2. MM/GBSA Calculations

Five thousand conformations from the last 8 ns of MD simulations were collected for MM/GBSA binding free energy calculations. The results are presented in Table 1. A more negative value of the ΔG_bind_ implies a favorable binding of the ligand to the enzyme. According to the values of ΔG_bind_, the ternary complex of mtbDHFR:NADPH with P169 and DHF was more favorable than the complex with either P157 or P1.

The majority of favorable binding of P169 and DHF is contributed from ΔE_ele_ of carboxylate anions (R-COO^−^). The ΔE_vdw_ and ΔG_SA_ are significant to the binding affinity of P169 for mtbDHFR. The TΔS indicates the positional change of the system. As shown in Table 1, the mtbDHFR in the complex with either P169 or DHF had a large entropy change, approximately −30 kcal/mol. The results indicate the high order of the systems due to the strong binding of P169 or DHF molecules to the binding site of mtbDHFR. The MD simulations show that the binding of all ternary mtbDHFR complexes in this study was energetically favorable, spontaneously occurring based on the negative sign of ΔG_bind_.

### 3.3. The Dynamics of Loops and Domains

**Figure 3:**
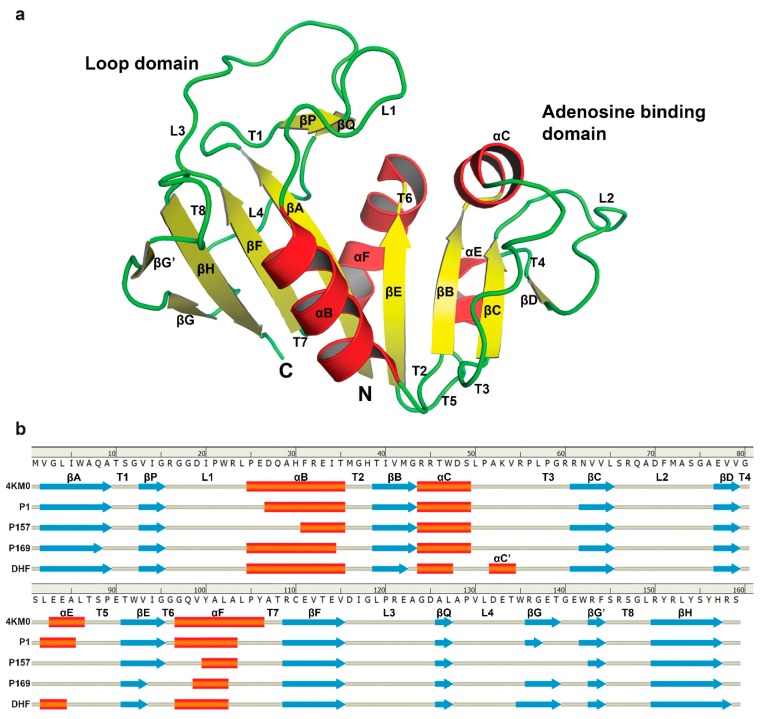
The schematic structure of mtbDHFR. (**a**) Structure of mtbDHFR. Coils in green, alpha-helices in red, and beta-strands in yellow; (**b**) Sequence and secondary structure of each MD simulation at 16 ns. The secondary structural elements are assigned according to the Define Secondary Structure of Proteins method (DSSP) by Discovery Studio Visualizer 4.0 program [14].

The secondary structure of mtbDHFR in all complexes exhibited the same general folding, as shown in Figure 3a. The nomenclature of secondary structural elements is the same as those defined by Li et al. [27], as shown in Figure 3b.

**Figure 4:**
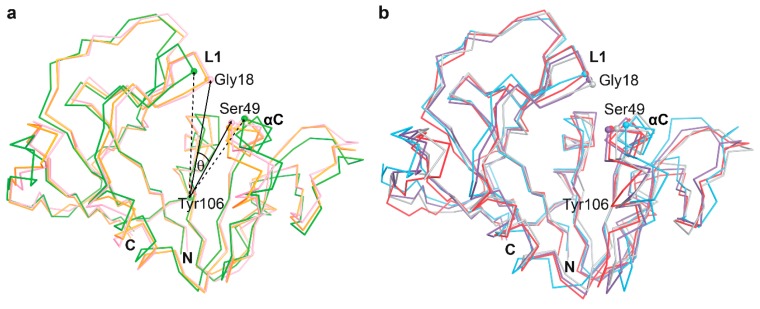
Overall structural comparison of mtbDHFR. (**a**) Superposition of the binary mtbDHFR:NADPH crystal structures in closed (4KL9 in pink and 1DG8 in orange) and open (4KLX in green) conformation states. The angle θ is measured from the angle between three alpha-C atoms (ball models) of Ser49, Tyr106, and Gly18; (**b**) Superposition of the ternary mtbDHFR:NADPH:2,4-diaminopyrimidine complex from MD simulations at 16 ns: P1 (blue), P157 (purple), P169 (red), and DHF (gray).

The available crystal structures of mtbDHFR in RCSB PDB are reported in two conformations, corresponding to “open” and “closed” forms (Table 2). Loop L1 of mtbDHFR corresponds to loop Met20 of *E. coli*, and the fluctuation of this loop causes the different conformations for DHFR [12]. The largest difference between open and closed conformations of mtbDHFR occurs between the Gly18 on loop L1 (residues 16–24) and Ser49 on helix αC (residues 44–49). To distinguish the open and closed conformations of mtbDHFR in this study, the θ angle is defined, which is measured from the three alpha-C atoms of the three amino acid residues Ser49, Tyr106, and Gly18 (Figure 4). The superposition of the open and closed forms of the binary mtbDHFR:NADPH complexes are presented in Figure 4a. According to the θ angle values of nine crystal structures, the average value of θ for the closed conformation was 14.74 ± 1.68 degrees, and the open conformation was 18.41 ± 1.17 degrees.

**Figure 5:**
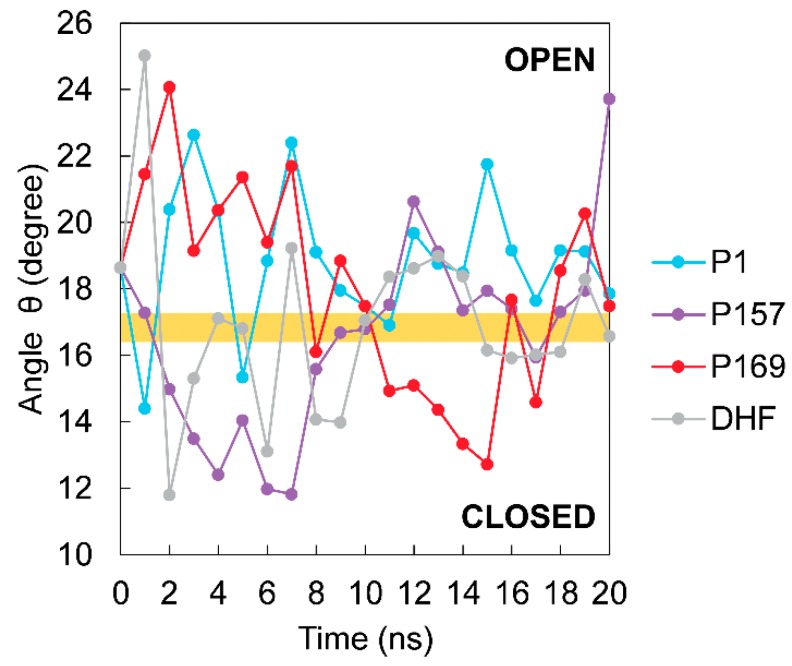
θ angle of mtbDHFR conformation at every 1-ns time interval of each MD simulation system: P1 (blue), P157 (purple), P169 (red), and DHF (gray). The orange shading indicates the upper bound and lower bound of closed and open conformations.

Arora’s group reported that three conformations of *E. coli* DHFR:NADPH:DHF change during the catalysis pathway, which are closed, open, and occluded, depending on the position of the Met20 loop [28] that corresponds to loop L1 in mtbDHFR. Dynamics of loop fluctuation of *E. coli* DHFR were observed in a wide range of time-scales, ranging from picoseconds to milliseconds [14]. Therefore, 20 ns of MD simulation should be sufficient to monitor the loop dynamics of mtbDHFR that is involved in the binding of ligands, including the inhibitors, substrate, and cofactor. The motions of loop L1 of mtbDHFR for each MD system were measured during 20 ns. The motion of loop L1 of mtbDHFR:NADPH:DHF in our MD system is indicated by θ angle (Figure 5, gray line). The graph demonstrates that at the last 8 ns of MD simulations, the loop L1 of mtbDHFR:NADPH complexes with either P1 (blue) or P157 (purple) is mostly open, whereas in the complexes with P169 (red) and DHF (gray) the loop is frequently closed. The change of loop L1 to a closed conformation also occurs in the *E. coli* DHFR catalytic cycle, as reported by Sawaya and Kraut. The conformation changes of the loops depend on the ligands bound to the active site [15]. This may explain why P169 has a stronger binding affinity for mtbDHFR, as compared to P1 and P157. Moreover, compound P169 and substrate DHF showed strong binding affinities for mtbDHFR:NADPH, as indicated by the low ΔG_bing_ in Table 1. The results agree with the previous report of *E. coli* DHFR, that the closed conformation is thermodynamically most favored [28] and has a stronger interaction with protein-ligand complexes.

### 3.4. The Binding of NADPH on Mycobacterium tuberculosis Dihydrofolate Reductase

**Figure 6:**
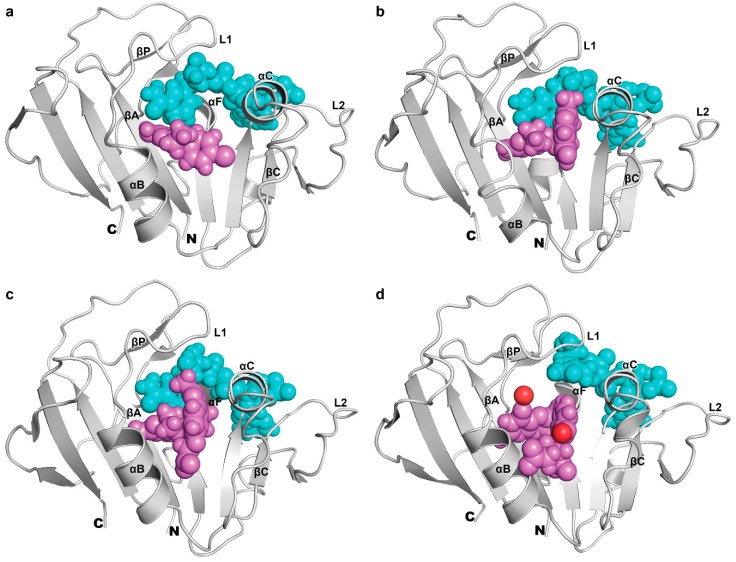
NADPH (blue) and ligand (pink) binding sites on mtbDHFR (gray ribbon) for MD simulations at 16 ns: (**a**) P1; (**b**) P157; (**c**) P169 and (**d**) DHF. Two water molecules (red spheres) are found in the substrate DHF binding site.

The cofactor NADPH is a significant factor that assists the binding between a protein and ligand. The conformations of NADPH in each MD simulation at 16 ns were chosen as a representative to study the binding behaviors of NADPH in each system. The median of the last 8 ns of the simulation period is shown in Figure 6. The NADPH molecule (see Figure 1e) consists of “adenine ribose-5-phosphate” and “nicotinamide ribose” moieties, which are connected by diphosphate at the middle. The structure alignment of the NADPH molecule in each ternary complex is almost identical. The adenine ribose-5-phosphate and diphosphate moieties of NADPH in all systems stay in the same region and have a similar configuration, except the “nicotinamide ribose” moiety. The adenine ribose-5-phosphate of NADPH binds to the C-terminus of βC, N-terminus of αC, and L2 of mtbDHFR. The adenine moiety has also shown interactions with Leu65 (βC), Arg67 (L2), and Val99 (αF) via hydrophobic interactions. The ribose-5-phosphate of NADPH forms H-bonds to Arg44, Ser66, and Arg67. The diphosphate group in the middle part of NADPH binds near the helices αC and αF and forms many hydrogen bonds. One significant difference observed between the structure of NADPH on mtbDHFR in a complex with inhibitors and substrate is its nicotinamide ribose moiety. In inactive ternary complexes (P1, P157, and P169), the nicotinamide ribose of NADPH forms H-bonds with Ala7 and Ile14 on βA and βP, respectively. Due to the occurrence of the hydrogen bond with Ser49 on αC, the nicotinamide ribose moiety of an active ternary complex has DHF, which bends outward from the binding site and moves closer to the αC helix (Figure 6d).

### 3.5. Binding of Substrate and Inhibitor to Mycobacterium tuberculosis Dihydrofolate Reductase

**Figure 7:**
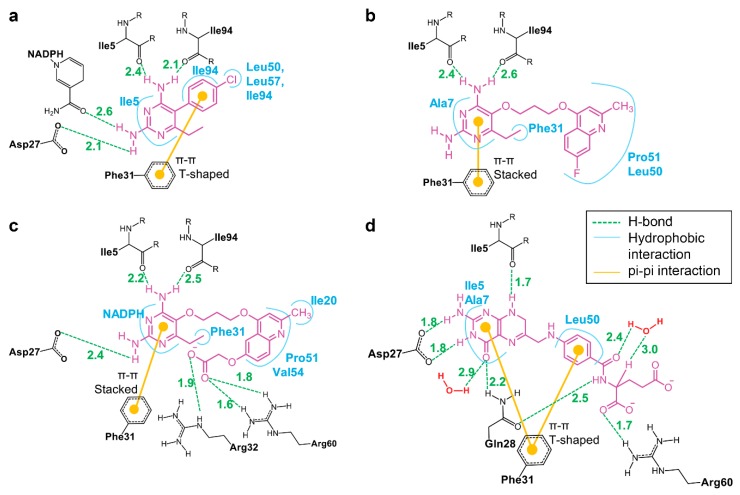
Interactions between amino acid residues in the mtbDHFR binding site with the ligands: (**a**) P1; (**b**) P157; (**c**) P169; and (**d**) DHF from the MD simulations. The distances (in Å) of hydrogen bonds are represented by green dashed lines. The aromatic (π-π) interactions are represented by yellow lines, and the hydrophobic interactions are represented by blue lines.

**Figure 8:**
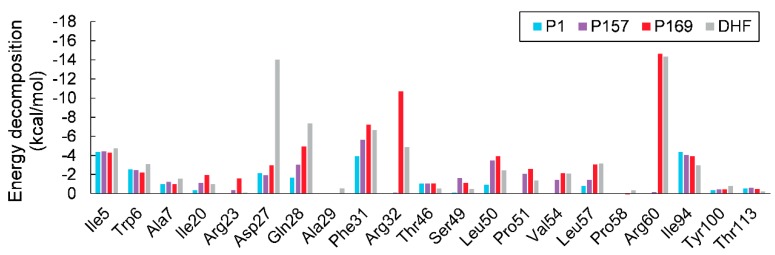
Energy decomposition of amino acid residues in the binding site towards the ligand in each ternary complex: P1 (blue bars), P157 (purple bars), P169 (red bars), and DHF (gray bars).

Interactions of substrate or inhibitors with the binding site of mtbDHFR at 16-ns MD simulations are shown in Figure 7. The substrate DHF, located at the active site on mtbDHFR, is directed by the strong hydrogen bonds between hydrogen atoms from the amino groups of the substrate’s pteridine ring with the carbonyl oxygen of Ile5 and the carboxylic group Asp27 residues. Pairwise energy decomposition of the same selected interval as that used in the free binding energy calculation was used to investigate the significance between the interaction of amino acid residues and the compounds or the substrate in each system, as depicted in Figure 8. Two hydrogen bonds between the amine and the backbone carbonyl groups of DHF and Gln28 residue on αB (Figure 7d, green lines) make the DHF molecule stay in the pucker V shape, as seen in Figure 6d. Two water molecules, found in the active site, form hydrogen bonds with the carbonyl oxygens of the pterin ring and the pABG groups of DHF molecule (Figure 1). The pucker V shape of the DHF molecule is also stabilized by the π-π interactions of its pteridine ring and its benzyl ring with the phenyl ring of the Phe31 residue (Figure 7d, yellow lines). The strong hydrogen bonds between the O-carboxylate of DHF and the H-amine of Arg60 residues (Figure 7 and Figure 8, gray bars) enhance the binding affinity between them.

Three inhibitors (P1, P157, and P169) are located at the same binding site of DHF on mtbDHFR (Figure 6). The pyrimidine ring of inhibitors is held in the interior of a deep cleft through hydrogen bonds and van der Waals interactions, as seen in Figure 7a–c. The 2,4-diamino groups interact with the carbonyl groups of three amino acid residues: Ile5, Ile94, and Asp27, via hydrogen bonding. The π-π interactions between the pyrimidine ring of inhibitors and the phenyl ring of Phe31 contribute to stabilize the complex conformation (Figure 8). Van der Waal interactions between the substituent at C5 on the pyrimidine ring of inhibitors with amino acid residues 50–54 on mtbDHFR are present (Figure 7, blue lines and Figure 8). These interactions result in the loss of the spiral conformation (αC’) of the enzyme, which is in the mtbDHFR:NADPH:DHF configuration (as represented in Figure 3b), and cause the P157 and P169 molecules to possess a pucker V shape (Figure 6b,c). The substitution at C5 on the pyrimidine ring of inhibitor molecules with the propoxy-(fluoro-methylquinolin) moiety in P157 and with the propoxy-(carboxylatemethoxy-methylquinolin) moiety in P169 increases the stability of the ternary complex systems and significantly improves their antifolate efficiency, as compared to P1 where the parent molecule has a rigid chlorophenyl substituent at C5. Moreover, the hydrogen bonds between the O-carboxylate of P169 with the H-amine of Arg32 and Arg60 residues (Figure 7c and Figure 8, red bars) enhance the binding between them, which explains the low value of ΔG_bind_ for this system as well as the high antifolate efficiency of this compound.

The inhibitors bind preferentially to the binary form of the enzyme with NADPH, which is the binding site of the substrate DHF [29,30]. The simulations indicate that P1, P157, and P169 are competitive substrate inhibitors, which bind to the same binding site of DHF in mtbDHFR. The substitution at C5 on the diaminopyrimidine moiety of the molecule with different functional groups yields significant changes for the antifolate affinity of compounds as well as their orientations in the binding pocket.

## 4. Conclusions

From the MM/GBSA calculations, it was shown that changes in the electrostatic and van der Waals energy play a major role in the binding of potent compound P169 and substrate DHF. P169 has a strong binding with the enzyme, which is similar to the natural substrate DHF and decreases the disorder of the system. Moreover, the enzyme mtbDHFR is preferred to adopt the closed conformation of loop L1 when binding to P169 or DHF. L1 of mtbDHFR in non-potent compound P1 mostly has an open conformation along the simulation, as a result of the lower binding affinity between the P1 and mtbDHFR. The active ternary complex of mtbDHFR:NADPH:DHF forms strong bonds to the enzyme via many H-bonds and hydrophobic interactions. Because DHF is a natural substrate of DHFR, this molecule therefore exhibits highly precise binding into the binding pocket of the enzyme via hydrogen bonding to Asp27, Gln28, Arg32, and Arg60 of mtbDHFR. In inactive ternary complexes, where the enzyme binds to the inhibitor, the amino groups (NH_2_) of all 2,4-diaminopyrimidine compounds exhibit highly specific binding to the Ile5 and Ile94 amino acid residues via hydrogen bonds. The different substitution at C5 of the compounds affects the binding affinities for the enzyme. Non-potent compound P1 is rigid and short, while potent compound P157 and P169 have a flexible and long substituent at C5 of the pyrimidine ring, which promotes the strong binding affinity for the enzyme. Moreover, P169 has mimicked the ability of the binding of DHF through the amino groups from 2,4-diaminopyrimidine and the carboxylate group from the substitution at C5. Therefore, the P157 and P169 are potent and can be developed as inhibitors of mtbDHFR in the future.

## Figures and Tables

**Table 1:** The average binding free energy (ΔG_bind_) and the energy components for each mtbDHFR-NADPH-ligand complex from Molecular Mechanics/Generalized Born Surface Area (MM/GBSA) calculations.

Energy (kcal/mol)	P1	P157	P169	DHF
ΔE_vdw_	−31.93	−46.23	−52.60	−46.65
ΔE_ele_	−6.77	−14.98	−85.70	−127.65
ΔG_GB_	19.83	32.78	86.52	128.08
ΔG_SA_	−3.96	−5.51	−7.09	−6.44
ΔE_MM_	−38.71	−61.21	−138.31	−174.30
ΔG_solv_	15.87	27.27	79.44	121.63
TΔS	−21.48	−26.04	−30.27	−30.17
ΔG_bind_	−1.36	−7.90	−28.60	−22.50

ΔE_vdw_: van der Waals energy, ΔE_ele_: electrostatic energy, ΔG_GB_: electrostatic solvation energy, ΔGSA: non-electrostatic solvation component, ΔE_MM_: molecular mechanics energy, ΔG_solv_: solvation free energy, TΔS: conformational entropy change.

**Table 2:** The conformation type and the θ angle of 9 crystal structures of mtbDHFR.

Complex	PDB ID	Conformation	θ Angel (Degrees)
mtbDHFR-NADPH	4KLX	open	18.34
mtbDHFR-NADPH	4KL9	closed	13.57
mtbDHFR-NADPH	1DG8	closed	15.07
mtbDHFR-NADPH-Methotrexate	1DF7	closed	15.81
mtbDHFR-NADPH-Br-WR99210	1DG7	closed	14.72
mtbDHFR-NADPH-Trimethoprim	1DG5	closed	14.54
mtbDHFR-NADPH-Trimethoprim	4KM2	open	18.79
mtbDHFR-NADPH-Cycloguanil	4KNE	open	17.88
mtbDHFR-NADPH-Pyrimethamine	4KM0	open	18.64

PDB ID: Protein Data Bank ID.
